# Multicentric real world evidence with palbociclib in hormone positive HER2 negative metastatic breast cancer in Indian population

**DOI:** 10.1038/s41598-021-95758-1

**Published:** 2021-08-10

**Authors:** Chaturbhuj Agrawal, Pankaj Goyal, Amit Agarwal, Rupal Tripathi, Chandragouda Dodagoudar, Saphalta Baghmar, Archana Sharma, Ullas Batra, Vineet Talwar, Sumit Goyal, Rajeev Kumar, Dinesh Chandra Doval

**Affiliations:** 1 Department of Medical Oncology, Rajiv Gandhi Cancer Institute and Research Centre, Sector-5, Rohini, New Delhi, India; 2 Department of Medical Oncology, Fortis Hospital, New Delhi, India; 3 Department of Research, Rajiv Gandhi Cancer Institute and Research Centre, New Delhi, India; 4 Department of Medical Oncology, BLK Super Specialty Hospital, New Delhi, India; 5 Department of Medical Oncology, Max Super Specialty Hospital, New Delhi, India; 6 Department of Surgical Oncology, Rajiv Gandhi Cancer Institute and Research Centre, New Delhi, India

**Keywords:** Medical research, Oncology

## Abstract

The combination of cyclin dependent kinase 4/6 inhibitors with endocrine therapy is the standard therapy in hormone receptor positive HER-2 negative metastatic breast cancer (HR+/HER2− MBC). Several randomized trials have shown the benefits of this combination, however, real world evidence in the Indian patients is warranted. The present study reports the largest real world multicentric data from Indian population on the use of Palbociclib in HR+/HER2− MBC. A multicentric study on the HR+/HER2− MBC patients who received palbociclib with hormonal agent (Aromatase inhibitors/Fulvestrant) between February 2017 and May 2020 was conducted. Clinical and demographic information and survival data was retrieved from the Hospital medical records. Among a total of 188 patients, 57% patients were premenopausal and 17% patients had bone only disease. Altogether, 115 (61%) patients received palbociclib with Aromatase inhibitors in the first line whereas 73 (39%) patients received it in the second line with Fulvestrant. The median follow up period with advanced disease was 13 months. The median progression free survival in the first line and second line was 20.2 months and 12 months, respectively (*p*-value < 0.0001). The objective response rate was 80% and 47.9% in first and second lines, respectively. Dose interruptions/ discontinuation were done in 14.9% and 2.7% patients in the first and second lines, respectively. In terms of toxicity, 10% patients had grade 3–4 adverse events. The present real world data of the use of palbociclib in Indian population suggests similar effectiveness to previously published real world evidences and has been adapted as the standard of care in the first and second line treatment of HR+/HER2− MBC.

Hormone receptor positive human epidermal growth factor 2 negative (HR+/HER2−) subtype is very common and for over a decade, hormonal therapies were the initial treatment of choice for metastatic breast cancer patients with this subtype^1^, even though it still remains very challenging to treat. However, endocrine resistance has marred the success rates of this treatment strategy^2^. This has in turn led to finding out newer inhibitors for this heterogeneous group of cancers.

The US food and Drug Administration (FDA) approved the use of three cyclin dependent kinase (CDK) inhibitors (palbociclib, ribociclib & abemaciclib) for the treatment of HR+/HER2− locally advanced or metastatic breast cancer^3–5^. The use of a selective cyclin dependent kinase (CDK) 4/6 inhibitor, Palbociclib in combination with an aromatase inhibitor as initial endocrine therapy or Fulvestrant following endocrine therapy has been approved in HR+/HER2− patients with advanced/metastatic breast cancer. The placebo controlled PALOMA-2 study showed a higher median progression free survival (PFS) for patients treated with Palbociclib and Letrozole (27.6 months) as compared to those with placebo and Letrozole (14.5 months). Thereafter, in the PALOMA-3 trial, patients with relapsed/ refractory HR+/HER2− advanced/ metastatic breast cancer, a higher PFS was noted in patients receiving Palbociclib and Fulvestrant (9.5 months) in comparison to those receiving Placebo and Fulvestrant (4.6 months)^6–8^.

Studies assessing the use of Palbociclib in combination with Letrozole/ Fulvestrant have been conducted in different countries^9–12^. However, till today, very little real world evidence is available from the developing countries like India with varied genetic and ethnic backgrounds and a relatively higher prevalence of the disease. The heterogenous structure of the Indian society has also led to variations in disease awareness, approach towards treatment, affordability etc. In turn, a rapid increase in the incidence of breast cancers in India may be caused due to socioeconomic factors including delayed marriage and child birth, lifestyle modifications, etc. More so, the demographic profile of the patients is also different from the western world^13^.

The present multicentric study was therefore conducted to evaluate the real world experience related to the treatment patterns and clinical and survival outcomes of HR+HER2− patients with advanced/metastatic breast disease receiving Palbociclib in combination with hormonal agent (aromatase inhibitor or Fulvestrant) in India.

## Material and methods

A multi centric retrospective study during the period February 2017 to May 2020 was conducted on the HR+/HER2− MBC patients who received palbociclib with hormonal agent (Aromatase inhibitors/ Fulvestrant) and patient data was collected from two hospitals namely Rajiv Gandhi Cancer Institute & Research Centre (RGCI&RC), Delhi and BLK super specialty hospital (BLK), New Delhi, India. RGCIRC and BLK contributed 132 and 56 patients, respectively. The study was approved by the Institutional Review Board, RGCI&RC (vide letter dated 05.03.2021) and Institutional Ethics Committee, BLK (vide letter dated 18.03.2021) and granted a waiver from the consenting process. The study was conducted as per the Helsinki Declaration.

Medical records were reviewed retrospectively of these patients. Information related to the demographic profile, tumor type, histopathology details, chemotherapy regimen, use of endocrine therapy, response and follow up information was recorded. The primary end point of the study was PFS whereas the secondary end point was overall survival (OS).

The TNM AJCC 7th Edition guidelines was followed for the staging of breast cancer^14^. Formalin fixed paraffin embedded sections were used for immunohistochemical staining and the expression levels of estrogen receptor (ER), progesterone receptor (PR) and human epidermal growth factor receptor 2 (HER2) were assessed and scored as per the ASCO-CAP guidelines 2007^15^.

### Palbociclib treatment plan

In the first line, letrozole 2.5 mg once daily and palbociclib 125 mg daily was given for 3 weeks followed by 7 days rest. Hematological examination was done on day 15 for the initial 2–3 cycles. In the second line, injection fulvestrant 250 mg deep intramuscularly was given on day 1, 14 and 28 followed by once in 28 days henceforth. Tablet palbociclib 125 mg per oral for 21 days was given followed by 7 days rest. Hematological examination was done on day 15 for the initial 2–3 cycles. The evaluation was done using CT scan of chest and abdomen and PET-CT scan at baseline and after every 3 cycles initially for 6 months followed by 6 monthly evaluation.

SPSS version 23 for Windows (SPSS Inc, Chicago IL, USA) was used for statistical analysis. Pearson χ2 or Fisher’s Exact Test, whichever appropriate, was used for categorical variables. Survival analysis was performed using the Kaplan Meier method^16^. Log Rank test was used to compare the difference in survival among the groups. A two sided *p*-value < 0.05 was considered as significant.

## Results

A total of 188 patients were included in the study. The median age of the patients was 58.5 years (32–85 years). Around 98% patients were females and majority of the patients were premenopausal (57.4%). The demographic profile of the patients is shown in Table 1. The proportion of patients presenting with disease in the right and left breast was also similar (48.4% and 48.9%, respectively) and 17% patients had bone only disease. Around 52% patients presented with metastatic disease at presentation in the hospital. Brain metastasis was reported in 4.8% patients and less than three metastatic sites were noted in 60.1% patients. Visceral metastasis was most commonly observed in 82.4% patients. All premenopausal women received ovarian suppression or ovarian ablation (OS/OA).

**Table 1:** Baseline characteristics (N = 188).

Variable	n (%)
Median age in years (range)	58.5 (32–85)
**Menopausal status**	
Pre	108 (57.4)
Post	80 (42.6)
**Sex**	
Transgender	1 (0.5)
Male	3 (1.6)
Female	184 (97.9)
**Laterality**	
Right	91 (48.4)
Left	92 (48.9)
Bilateral	5 (2.7)
**Stage at presentation**	
Early	45 (23.9)
Locally advanced	46 (24.5)
Metastatic	97 (51.6)
**Brain metastasis**	
Present	9 (4.8)
Absent	179 (95.2)
**Site of metastasis**	
Visceral	155 (82.4)
Bone only	32 (17)
Lymph node only	1 (0.5)
**Line of hormonal therapy**	
First line	115 (61.2)
Second line	73 (38.8)
**Prior chemotherapy before palbociclib in the neoadjuvant/adjuvant setting**	
Yes	95 (50.5)
No	87 (46.3)
Not available	6 (3.2)
**Response at first assessment**	
Complete response	9 (4.8)
Partial response	118 (62.8)
Stable disease	28 (14.9)
Progressive disease	33 (17.6)
**Dose interruption/reduction/discontinuation**	
No	146 (77.7)
Yes	28 (14.9)
Discontinued	9 (4.8)
Not available	5 (2.7)

In terms of therapy, Palbociclib was offered to all the patients with the disease condition, however, 25% patients had refused the same due to logistic reasons. The most common starting dose of Palbociclib was 125 mg/day. Palbociclib with first and second line hormonal therapy was given in 61.2% and 38.8% patients, respectively. Administration of prior chemotherapy before Palbociclib in the neoadjuvant/adjuvant setting was reported in 50.5% patients. Partial response at first assessment was most common and observed in 62.8% patients. Dose interruptions and discontinuation was done in 14.9% and 4.8% patients, respectively. In terms of toxicity, 10% patients had grade 3–4 adverse events and 20% patients had neutropenia grade 3–4 while no patient had febrile neutropenia.

Table 2 profiles the comparison of baseline and clinical characteristics on the basis of line of use of Palbociclib. On the basis of the stage of disease at presentation, 68.7% patients had metastatic disease at presentation in the patients who subsequently received Palbociclib + AI as compared to 24.7% metastatic patients received Palbociclib and Fulvestrant (*p*-value < 0.0001). A statistically significant association was also observed when comparisons were done related to prior chemotherapy before Palbociclib in the neoadjuvant/adjuvant setting and response at first assessment with the line of use of Palbociclib (*p*-value < 0.0001).

**Table 2:** Comparison of baseline and clinical characteristics on the basis of line of use of pablbociclib.

Variable	Palbociclib + AI N = 115	Palbociclib + fulvestrant N = 73	*p*-value
**Menopausal status**			
Pre/post	61/54	47/26	0.125
**Sex**			
Transgender/male/female	1/1/113	0/2/71	0.445
**Laterality**			
Right/left/bilateral	52/61/2	39/31/3	0.274
**Stage at presentation**			
Early/locally advanced/metastatic	16/20/79	29/26/18	** < 0.0001**
**Brain metastasis**			
Present/absent	7/108	2/71	0.295
**Site of metastasis**			
Visceral/bone only/lymph node only	99/15/1	56/17/0	0.145
**Prior chemotherapy before palbociclib in the neoadjuvant/adjuvant setting**			
Yes/no	41/17	54/16	** < 0.0001**
**Response at first assessment**			
CR/PR/SD/PD	7/85/16/7	2/33/12/26	** < 0.0001**
**Dose variation**			
No/yes/discontinued/not known	88/19/6/2	58/9/3/3	0.652

*p*-values in bold indicate statistical significance.

**Figure 1:**
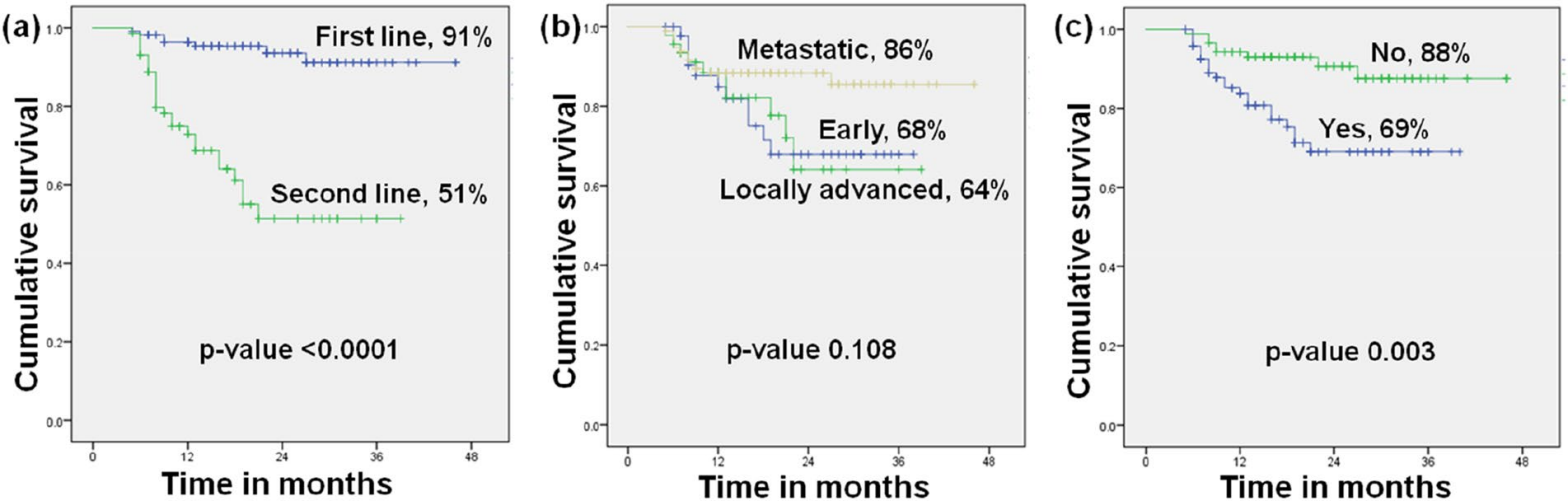
Three years progression free survival on the basis of (**a**) line of hormonal therapy (**b**) stage of disease at presentation (**c**) prior chemotherapy before palbociclib in the neoadjuvant/adjuvant setting.

The median follow up period was 17 months. The median PFS in first line was found to be 20 months while in second line it was 12 months (*p*-value < 0.0001). The objective response rate was 80% and 47.9% in first and second lines, respectively. Figure 1 shows the Three years progression free survival on the basis of (a) line of hormonal therapy—first line 91% vs. second line 51%, *p*-value < 0.0001 (b) stage of disease at presentation—early disease 68% versus locally advanced disease 64% vs. metastatic disease 86%, *p*-value 0.108 and (c) prior chemotherapy before palbociclib in the neoadjuvant/adjuvant setting—yes 69% versus no 88%, *p*-value 0.003.

## Discussion

The treatment strategy of breast cancers has been modified by the identification of different subgroups of this heterogenous disease and emergence of resistance to endocrine therapy till today remains a matter of grave concern in patients with HR+/HER2− advanced/metastatic breast cancer. Improved PFS with the use of hormonal therapy with CDK 4/6 inhibitor in comparison to chemotherapy or hormonal therapy alone in HR+/HER2− advanced/ metastatic breast cancer has been reported in a meta-analysis. Palbociclib in combination with either aromatase inhibitor or Fulvestrant has emerged as an effective treatment strategy in this setting^6–8^, however, real world data from the Indian population is lacking. Palbociclib is offered to all the eligible patients, however, around 25% patients had refused this drug due to logistic reasons and hence could not be included in the study. This is the largest study evaluating the experience with Palbociclib in India assessing the treatment and survival outcomes of advanced/ metastatic breast cancer patients.

In the real world setting in US, Argentina and Germany in the patients with HR+/HER2− advanced/metastatic breast cancer, the Ibrance Real World Insights (IRIS) study evaluated the use of palbociclib in these patients. In this retrospective chart review, patients who received palbociclib with either an aromatase inhibitor (AI) as initial endocrine-based therapy in postmenopausal women or fulvestrant-based therapy in women with disease progression following endocrine therapy were studied^9^. In stark contrast with the results of IRIS study in USA, the majority of the patients in this study were premenopausal (57.4%) as compared to 12.6% patients in their study. Viscera as the site of metastasis was observed in 82.4% and 46.4% patients in our and IRIS studies, respectively. In the PALOMA-2 trial, 23% patients had bony metastasis only as compared to a frequency of 17% observed in our study^6^. Also, prior chemotherapy before Palbociclib in the neoadjuvant/adjuvant setting had been administered to 50.5% patients in our study as compared to 19.8% patients in the IRIS study^9^.

It is important to note that the majority of the patients included in our study had locally advanced/metastatic disease at presentation (76.1%) as also observed in the IRIS study which had enrolled 79.3% patients with metastatic disease at presentation^9^. This is in contrast to the study by Fountzilas et al. in which the distribution of the patients in the different groups was more even^17^. Indian breast cancer patients present at a younger age with an advanced stage of the disease including larger tumor size, higher histopathological grade and more aggressive disease. Diversity of the unique genetic profile of the Indian population is also reflected in terms of patient population, sample size, disease characteristics etc. In a previous study from India on metastatic breast cancer patients, 60% of the patients were premenopausal^18^. A high proportion of Indian women present with denovo metastatic disease. This has been witnessed in our centre. There are no routine screening strategies in India and the patient needs to bear the cost of diagnosis and treatment of the disease. Factors such as socioeconomic status, educational background and marital status greatly affect the presentation of the disease. In a study from India, it has been reported that 60% of breast cancer presents present with stage 3 or 4 disease^19^. A study from northern India showed that TNM stage III was the commonest stage constituting 62% patients^20^ whereas a study from Western India reported a total of 54% patients with advanced stage of the disease^21^. This is in contrast to the western world where a study reported around 6% patients with advanced stage of the disease^22^.

Dose interruptions and discontinuation was done in 14.9% and 2.7% patients, respectively. However, another study from India has reported that dose reductions due to toxicities including fatigue or mucositis were required in 24% patients. In the IRIS study, adjustments in Palbociclib dosage were required in 17.3% patients. In the PALOMA trials, it was mandated that all the patients had to be administered an initial dose of 125 mg/day^23^.

Importantly, palbociclib with first and second line hormonal therapy was given in 61.2% and 38.8% patients, respectively. A few preclinical studies reported a synergistic anti-tumor activity between the use of hormonal therapy and CDK4/6 inhibition in hormone receptor positive breast cancer cell lines^24^. A randomized phase 2 trial in post menopausal women with HR+/HER2− advanced breast cancer who progressed on the same hormonal therapy reported an increase in the median PFS with the use of palbociclib with hormonal therapy (10.8 months) as compared to palbociclib alone (6.5 months)^25^. However, palbociclib has also shown single agent activity in endocrine resistant HR+/HER2− advanced breast cancer patients^26^.

Overall, a total of 127/188 patients showed objective responses whereas progressive disease was reported in 17.6% patients. The objective response rate was 80% and 47.9% in first and second lines, respectively. The comparative analysis of the response rates observed in the different studies has been tabulated in Table 3. In the IRIS study which recruited patients undergoing first line treatment with palbociclib and aromatase inhibitor for advanced/metastatic breast cancer, 79.5% patient achieved objective responses in comparison to the PALOMA-2 study which showed objective responses in 42% patients. In a small study from India conducted on 24 patients, the ORR was 38% among the 21 patients with evaluable disease^27^.

**Table 3:** Comparative analysis of different real world studies on palbociclib.

References	N	ORR overall	Palbociclib + aromatase inhibitor		Palbociclib + fulvestrant	
			n	ORR	n	ORR
Taylor-Stokes et al.^9^	652	–	360	79.5%	292	74%
Rauthan et al. ^29^	25	41.7%	16	–	9	–
Gnanaguru et al.^27^	24	38%	–	–	–	–
Lee et al. ^30^	169	–	145	39.6%	24	28.6%
Lin et al. ^31^	281	32%	233	36.5%	48	10.4%
Present study	188	67.6%	115	80%	73	47.9%

In a previous study from India, the median PFS was 18 months^27^ as compared to 24.9 months in the PALOMA-2 trial^28^. In the study by Fountzilas et al., the median PFS irrespective of the line of therapy was 13.5 months for the 361 patients included in the analysis^17^. In the present study, the median PFS in the first line and second line was 20.2 months and 12 months, respectively (*p*-value < 0.0001). Variations have been observed in the data related to the clinical outcomes which could be largely attributed to the population differences.

Limitations of the study include its retrospective nature, thus affecting the quality of the real world data. Also, in the developing countries like India, adverse events might have been under reported. It is also important to note that the translation of results obtained in the clinical trials into the day-to-day clinical practice is challenging because of the tightly controlled and rigorous time bound monitoring of patients in the trials which may not be possible in the everyday practice in the real world setting.

Overall, this is the largest real world data on the use of palbociclib in Indian population suggesting an effectiveness similar to the previously published real world evidences and is the standard of care in the first and second line treatment of HR+/HER2− MBC.
